# Diversity of virulence level phenotype of hypervirulent *Klebsiella pneumoniae* from different sequence type lineage

**DOI:** 10.1186/s12866-018-1236-2

**Published:** 2018-08-29

**Authors:** Qiucheng Shi, Peng Lan, Danyan Huang, Xiaoting Hua, Yan Jiang, Jiancang Zhou, Yunsong Yu

**Affiliations:** 1 Department of Infectious Diseases, Sir Run Run Shaw Hospital, College of Medicine, Zhejiang University, Hangzhou, China; 2 Key Laboratory of Microbial Technology and Bioinformatics of Zhejiang Province, Hangzhou, China; 3 Department of Critical Care Medicine, Sir Run Run Shaw Hospital, College of Medicine, Zhejiang University, Hangzhou, China

**Keywords:** Hypervirulent *K. pneumoniae*, *Galleria. mellonella* infection model, 50% lethal dose, Multilocus sequence typing, Virulence level

## Abstract

**Background:**

Hypervirulent *Klebsiella pneumoniae* (hvKP) is emerging around the Asian-Pacific region and it is the major cause of the community-acquired pyogenic liver abscesses. Multidrug-resistant hypervirulent *Klebsiella pneumoniae* (MDR-hvKP) isolates were reported in France, China and Taiwan. However, the international-ally agreed definition for hvKP and the virulence level of hvKP are not clear.

**Results:**

In this study, 56 hvKP isolates were collected from March 2008 to June 2012 and investigated by string test, capsule serotyping, multilocus sequence typing (MLST), virulence gene detection and serum resistance assay. Among the 56 *K. pneumoniae* isolates, 64.3% had the hypermucoviscosity phenotype, meanwhile, 64.3% were the K1 serotype and 19.6% were the K2 serotype. Within the K1 serotype, 94.4% were ST23, and within the K2 serotype, ST65, ST86 and ST375 accounted for the same percentage 27.3%. The serum resistance showed statistically normal distribution. According to the 50% lethal dose of *Galleria. mellonella* infection model, hvKP isolates were divided into high virulence level group and moderate virulence level group. The ability of each method evaluating the virulence level of hvKP was assessed using the area under the receiver operating characteristic curve.

**Conclusions:**

K1 ST23 *K. pneumoniae* was the most prevalent clone of the hvKP. However, K1 ST23 *K. pneumoniae* was the dominant clone in the moderate virulence level group. MLST was a relatively reliable evaluation method to discriminate the virulence level of hvKP in our study.

**Electronic supplementary material:**

The online version of this article (10.1186/s12866-018-1236-2) contains supplementary material, which is available to authorized users.

## Background

*Klebsiella pneumoniae*, a well-known enteric gram-negative bacillus, often causes hospital-acquired infections, such as pneumonia, urinary tract and bloodstream infections [1]. During the past two decades, a new hypervirulent variant of *K. pneumoniae* (hvKP) was reported. The hvKP can cause community associated liver abscesses, as well as possible complications including metastatic meningitis and endophthalmitis [2]. The hvKP was usually associated with hypermucoviscosity and antibiotic-sensitivity.

The hvKP can cause serious infections in ambulatory and healthy hosts and result in metastatic spread of infections [3]. The appearance of colonies grown on an agar plate is hypermucoviscous [4]. According to the genomic data, in addition to *iroBCD*, *iucABCD* and *rmpA/A2*, Ye et al. found 21 new genes to be new virulence factors of *K. pneumoniae*. Several of these genes had been proposed as virulence factors in other bacteria, such as the gene encoding SAM-dependent methyltransferase and *pagO* which protected bacteria from phagocytosis [5].

Moreover, multidrug-resistant hypervirulent *Klebsiella pneumoniae* (MDR-hvKP) isolates had been reported. A K2 ST86 hvKP strain producing CTX-M-3 was reported in France. A K1 hvKP strain producing CTX-M-15 was reported in Taiwan. One K2 ST65 and five K1 hvKP carbapenem-resistant strains were reported in China [6–9].

Different terms of hvKP were used by investigators as there was no international-ally agreed definition. Basically, the definition of hvKP was based on the clinical manifestations, such as liver abscesses, metastatic meningitis and endophthalmitis [4]. Considering the life-threatening nature of liver abscess and the diversity of the patients’ prognosis, discovering a predictable phenotype or gene to identify hvKP and the virulence level of hvKP is very critical. In this study, we determined the string test, serotyping, multilocus sequence typing, serum resistance and virulence-associated genes of 56 hvKP isolates. The virulence level of these isolates were verified by the LD_50_ of *Galleria. mellonella* infection model. Meanwhile, we compared the discriminating power of each method by the area under the receiver operating characteristic (AUROC) curve, the bigger AUROC was associated with better discriminative power.

## Results

*G. mellonella* haemolymph contains haemocytes, which function in a manner similar to phagocytes in mammals [10], therefore, the LD_50_ of *G. mellonella* larvae is a method used to verify the virulence level of hvKP. The LD_50_ values of all the isolates were distributed from 3.06 to 5.84 log_10_ CFU/ml. To analyse the characteristic phenotype and genes associated with virulence level, all the 56 isolates were separated into two groups, according to the density graph (Additional file 1: Figure S1). Accordingly, 27 isolates with an LD_50_ less than 5.06 log_10_ CFU/ml were defined as the high-virulence level (HVL), while the other 29 isolates fell into the moderate-virulence level (MVL) group. The results of statistical analyses between HVL and MVL groups were shown in Table 1.

**Table 1:** The results of statistical analyses between high virulence level and moderate virulence level groups

	HVL(*n* = 27)	MVL(*n* = 29)	*p*-value
String Test	19 (70.4%)	17 (59.6%)	0.4123
Serotype			0.0112*
K1	12 (44.4%)	24 (82.8%)	
K2	8 (29.6%)	3 (10.3%)	
MLST			
ST23	12 (44.4%)	22 (75.9%)	0.0277*
Serum Resistance	49.9 ± 0.72%	50.1 ± 0.72%	0.7779
Virulence-associated genes			
*magA*	12 (44.4%)	24 (82.8%)	0.0048*
*rmpA*	26 (96.3%)	29 (100%)	0.4821
*kfu*	27 (100%)	29 (100%)	NA
*fimH*	27 (100%)	29 (100%)	NA
*wabG*	27 (100%)	29 (100%)	NA
*uge*	17 (63.0%)	23 (79.3%)	0.2397
*iroN*	27 (100%)	28 (96.6%)	> 0.9999
*iutA*	25 (92.6%)	26 (89.7%)	> 0.9999
*allS*	14 (51.9%)	24 (82.8%)	0.0214*
*entB*	27 (100%)	28 (96.6%)	> 0.9999

*p < 0.05

To investigate whether the hypermucoviscosity phenotype can determine the virulence level of hvKP, all the isolates were verified by the string test. Among the 56 hvKP isolates, 36 isolates (64.3%) showed hypermucoviscosity. In the hypermucoviscosity isolates, 100% were *rmpA* positive, whereas in non-hypermucoviscosity isolates, 95% were *rmpA* positive (Fisher’s exact test, *p* = 0.3571). By verifying the virulence level, 70.4% (19/27) had the hypermucoviscosity phenotype in the HVL group and 59.6% (17/29) in the MVL group had the hypermucoviscosity phenotype. The distribution of the hypermucoviscosity phenotype was compared between the two groups, and there was no statistically significant difference (Fisher’s exact test, *p* = 0.4123).

To determine the relationship of serotype and MLST among hvKP, we serotyped the isolates and found that 64.3% (36/56) of the isolates were the K1 serotype, 19.6% (11/56) were the K2 serotype, and the rest were the non-K1/K2 serotype. While the dominant sequence type was ST23 in the K1 serotype (34/36), and the sequence type in the K2 serotype distributed more dispersedly. Within the K2 serotype, ST65, ST86 and ST375 shared the same percentage 27.3% (3/11), with others included one ST374 isolate and one ST380 isolate (Table 2). In the HVL group, 44.4% (12/27) of the isolates were the K1 serotype, and 29.6% (8/27) were the K2 serotype. In the MVL group, however, 82.8% (24/29) of isolates were the K1 serotype, and 10.3% (3/29) were the K2 serotype (Chi-square, *p* = 0.0112). Likewise, in the HVL group, 44.4% (12/27) of isolates were ST23, and in the MVL group, 75.9% (22/29) of isolates were ST23 (Fisher’s exact test, *p* = 0.0277). The distributions of serotype and sequence type between the HVL and MVL groups were statistically significantly different, respectively.

**Table 2:** Serotype and MLST of 56 hypervirulent *Klebsiella pneumoniae* isolates

Serotype	MLST	Isolates	Total
K1	ST23	34 (94.4%)	36 (64.3%)
	ST367	1 (2.8%)	
	ST700	1 (2.8%)	
K2	ST65	3 (27.3%)	11 (19.6%)
	ST86	3 (27.3%)	
	ST375	3 (27.3%)	
	ST374	1 (9.1%)	
	ST380	1 (9.1%)	
Non-K1/K2	ST25	2 (22.2%)	9 (16.1%)
	ST29	2 (22.2%)	
	ST660	2 (22.2%)	
	ST806	1 (11.1%)	
	ST1049	1 (11.1%)	
	ST1764	1 (11.1%)	

64.3% *K. pneumoniae* isolates were the K1 serotype, 19.6% were the K2 serotype, and the rest were the non-K1/K2 serotype. The dominant sequence type was ST23 in the K1 serotype (34/36). Within the K2 serotype, ST65, ST86 and ST375 accounted for 27.3% of isolates, respectively. Others included one ST374 isolate and one ST380 isolate

To evaluate the viability of hvKP in foetal bovine serum (FBS), a serum resistance test was performed. The viability of hvKP in FBS reflected its virulence level, the lower the value of serum resistance, the higher the proportion of bacteria surviving. The serum resistance of 56 hvKP isolates was distributed from 42.4 to 59.8%, and showed statistically normal distribution (Shapiro-wilk normality test, *p* = 0.4354). By verifying the virulence level, the serum resistance of the 56 hvKP isolates showed that the diversity of serum resistance between the two groups was not significantly different (Unpaired *t* test, *p* = 0.7779).

To determine the connection between the virulence level of hvKP and virulence-associated genes, *magA*, *rmpA*, *kfu*, *fimH*, *wabG*, *uge*, *iroN*, *iutA*, *allS* and *entB* were screened. Genes such as *kfu*, *fimH* and *wabG* existed in all the 56 isolates, while *entB*, *iutA*, *iroN*, *rmpA* were detected in more than 90% of the isolates. For *magA*, 44.4% (12/27) of the isolates in the HVL group were positive and 82.8% (24/29) in the MVL group were positive, which showed a statistically significant difference between the two groups (Fisher’s exact test, *p* = 0.0048). Regarding *allS*, in the HVL group, 51.9% (14/27) of isolates were positive, but in the MVL group, 89.7% (26/29) of isolates were positive, which showed a statistically significant difference between the two groups (Fisher’s exact test, *p* = 0.0214). There was no statistically significant difference between the two groups in genes like *rmpA*, *uge*, *iutA*, *iroN* and *entB*. For example, 92.6% (25/27) of the isolates in the HVL group and 89.7% (26/29) of the isolates in the MVL group were *iutA* positive (Fisher’s exact test, *p* > 0.9999).

**Fig. 1:**
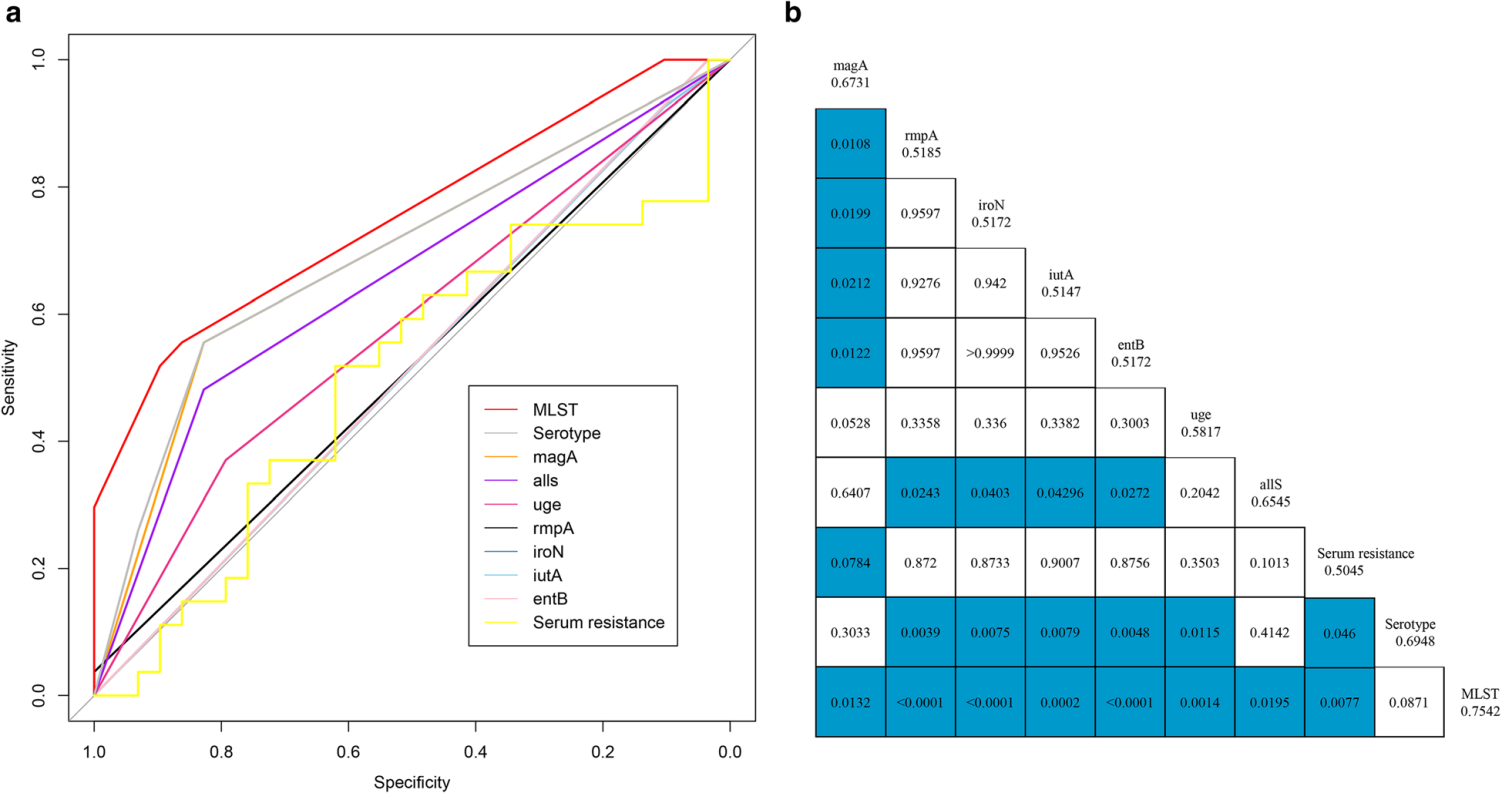
Receiver operating characteristic curves for the methods. **a** Receiver operating characteristic curves for *magA* (AUROC 0.6731, 95% CI 0.5542–0.7919), *rmpA* (AUROC 0.5185, 95% CI 0.4822–0.5548), *iroN* (AUROC 0.5172, 95% CI 0.4834–0.5510), *iutA* (AUROC 0.5147, 95% CI 0.4391–0.5903), *entB* (AUROC 0.5172, 95% CI 0.4834–0.5510), *uge* (AUROC 0.5817, 95% CI 0.4624–0.7011), *allS* (AUROC 0.6545, 95% CI 0.5357–0.7733), serum resistance (AUROC 0.5045, 95% CI 0.3475–0.6614), serotype (AUROC 0.6948, 95% CI 0.5745–0.8150), and MLST (AUROC 0.7542, 95% CI 0.6459–0.8625). **b** The results of statistical analysis and the value of AUROC for different methods. The blue bars mean *p* value < 0.05

To evaluate the discriminative power of all the methods above, receiver operating characteristic (ROC) curves were performed. MLST was found to be a relatively reliable evaluation method to determine the virulence level of hvKP (AUROC 0.7542, 95% confidence interval [95% CI] 0.6459–0.8625), followed by serotype (AUROC 0.6948, 95% CI 0.5745–0.8150) and *magA* gene (AUROC 0.6731, 95% CI 0.5542–0.7819) (Fig. 1a). The AUROC results of all the methods were shown in the Fig. 1b. Statistical analysis showed a significant difference between MLST and *magA* (*p* = 0.0132).

## Discussion

According to Fang et al., the string test, which reflects the hypermucoviscous phenotype, is the preferable laboratory-based surrogate marker for hvKP presently available and *rmpA* is important for hypermucoviscosity phenotype [11]. However, in this study, 95% of non-hypermucoviscosity isolates were *rmpA* positive, evaluated that *rmpA* gene is not sufficient for hypermucoviscosity phenotype. Second, the hypermucoviscosity phenotype was not statistically different between the two virulence level groups. Therefore, the string test is not a sensitive method to assess virulence level in hvKP. This is in agreement with a recent review, which suggests that hypermucoviscosity is not an appropriate phenotype of hypervirulence, and the hypermucoviscosity of *K. pneumoniae* is not equal to hvKP [12].

The K1 serotype of *K. pneumoniae* isolates contributed to 64.3% of hvKP, which was higher than the previous report in mainland China (39.2% and 43%) [13, 14]*.* The prevalence of the K1 serotype was statistically higher than the K2 and non-K1/K2 serotype in this study. Our MLST data matched the Bialek-Davenet et al. report, wherein most isolates of serotype K1 belonged to Clone Group 23 (CG23), and STs associated with serotype K2 were distributed into 3 main CGs: CG86, CG375, and CG380 [15]. In summary, the K1 ST23 isolates were the dominant clone of hvKP in China.

The K1 ST23 isolates occupied a higher proportion of the MVL group than the K2 serotype, which suggested that the K1 ST23 isolates had a moderate virulence level among the hvKP isolates. Compared to the AUROC of serotype, the AUROC of MLST showed a bigger area, which suggested that MLST was a relatively reliable method to evaluate the virulence level of hvKP. Further research will be needed to determine whether K1 ST23 isolates are in moderate virulence level than the other *K. pneumoniae* serotypes.

Virulence-associated genes were great for evaluating the hvKP, and some of the virulence-associated genes generally existed in the isolates of this study [13, 16]. In this study, virulence factors (*magA*, and *allS*) were highly associated with virulence level and were more prevalent in the MVL group. Other virulence factors showed no association with virulence level. Because of the complexity of the genetic background in clinical isolates, virulence-associated genes may not be a good choice to verify the virulence level of hvKP.

## Conclusion

The string test, serum resistance assay, virulence-associated genes, serotype, and MLST are determined in hvKP isolates. In summary, the string test and serum resistance assay are not proper methods to assess hvKP. The virulence level of these isolates are diverse. The LD_50_ of *G. mellonella* is probably suitable for verifying the virulence level, but it is not simple or convenient. MLST is relatively reliable to evaluate the virulence level of hvKP at present. Clinical infections are complicated and determined by both the bacteria and the host. K1 ST23 hvKP isolates are dominant in the Asian-Pacific region. Our data indicates that the K1 ST23 serotype isolates are in relatively moderate virulence level.

## Methods

### Collection and identification of *K. pneumoniae* clinical isolates

From March 2008 to June 2012, a total of 56 isolates of *K. pneumoniae* were isolated from patients with liver abscess in the first affiliated hospital of Zhejiang University. These isolates were defined as hvKP according to Shon and Russo [17].The *K. pneumoniae* isolates included in this study were identified by 16S rRNA Sanger sequencing and the primers used were 27F (*AGAGTTTGATCCTGGCTCAG*) and 1492R (*TACGGCTACCTTGTTACGACTT*).

### Hypermucoviscosity phenotypical identification of *K. pneumoniae* isolates

The hypermucoviscosity phenotypes of *K. pneumoniae* isolates were determined by the string test [18]. Briefly, a positive string test is the formation of a viscous string > 5 mm in length when a colony is grown on a blood agar plate at 37 °C overnight and stretched by an inoculation loop.

### Serum resistance assay

The serum resistance of each bacteria was determined by the method introduced by Mu et al. [19]. Briefly, four independent cultures per strain were grown overnight in Mueller–Hinton (MH) broth, diluted 1:1000 [MH broth or 20% foetal bovine serum (FBS) MH Broth] and aliquoted into a honeycomb 100-well plate in three replicates. The plate was agitated at 37 °C. The OD600 of each culture was determined every 5 mins for 16 h using the Bio-Screen (BioScreen Testing Services, Inc., Finland). The growth rate was estimated by an R script based on the OD600 curves [20]. The results were expressed as the ratio of the growth rate in 20% FBS MH broth and MH broth.

### Detection of serotype, virulence-associated genes and multilocus sequence typing (MLST)

In the project, serotype-specific genes for the K1 and K2 capsular serotypes and virulence-associated genes including *magA*, *rmpA*, *kfu*, *fimH*, *wabG*, *uge*, *iroN*, *iutA*, *allS* and *entB* were all amplified by polymerase chain reaction (PCR). The primers used were shown in the Additional file 2: Table S1. MLST of *K. pneumoniae* was performed as previously described (http://bigsdb.pasteur.fr/klebsiella/primers_used.html).

### 50% lethal dose (LD_50_) of *G.mellonella* infection model

*G. mellonella* larvae were acquired from an insect mass-rearing plant in Shandong and kept in darkness at 4 °C. They were used within 12 days since reception. For LD_50_ experiments, a series of 10-fold serial dilutions of overnight incubation bacteria were diluted in PBS, of which the CFU was approximately 10^9^ per millilitre. Then serial dilutions of *K. pneumoniae* in PBS were injected into *G. mellonella* larvae [21]. Ten larvae were injected at each dilution and incubated at 37 °C in the darkness. The dead larvae were counted for three days after injection. For each isolate, the LD_50_ was calculated according to the probit model, and the results were expressed as log_10_ LD_50_ [22].

### Statistical analysis

The Shapiro-Wilk normality test was performed to test the normality of the distribution of quantitative variables. For categorical variables, comparisons were performed by Chi-square analysis or Fisher’s exact test. All the quantitative variables that were normally distributed were presented as mean ± SD and compared by *t*-test. A *p* value < 0.05 was considered statistically significant. The ability of each method to discriminate the virulence level of *K. pneumoniae* was assessed by the area under the receiver operating characteristic (AUROC) curve, and the discriminative ability of the AUROC curves were compared by Delong’s test. The statistical software used was Prism5 (GraphPad Software, lnc.) and R 3.3.3 (pROC packages) for Windows.

## Additional files

Additional file 1: **Figure S1.** The density graph of LD_50_ of 56 hypervirulent *Klebsiella pneumoniae* isolates. (PDF 481 kb)

Additional file 2: **Table S1.** PCR primers used for studying Serotype and Virulence-Associated Genes of *Klebsiella pneumoniae* isolates. (PDF 481 kb) (DOCX 27 kb)
