# Supplementary figures and images for: Diversity of virulence level phenotype of hypervirulent Klebsiella pneumoniae from different sequence type lineage
Source: BMC Microbiol. 2018 Aug 29;18:94. doi: 10.1186/s12866-018-1236-2 (PMC6116568; doi:10.1186/s12866-018-1236-2)

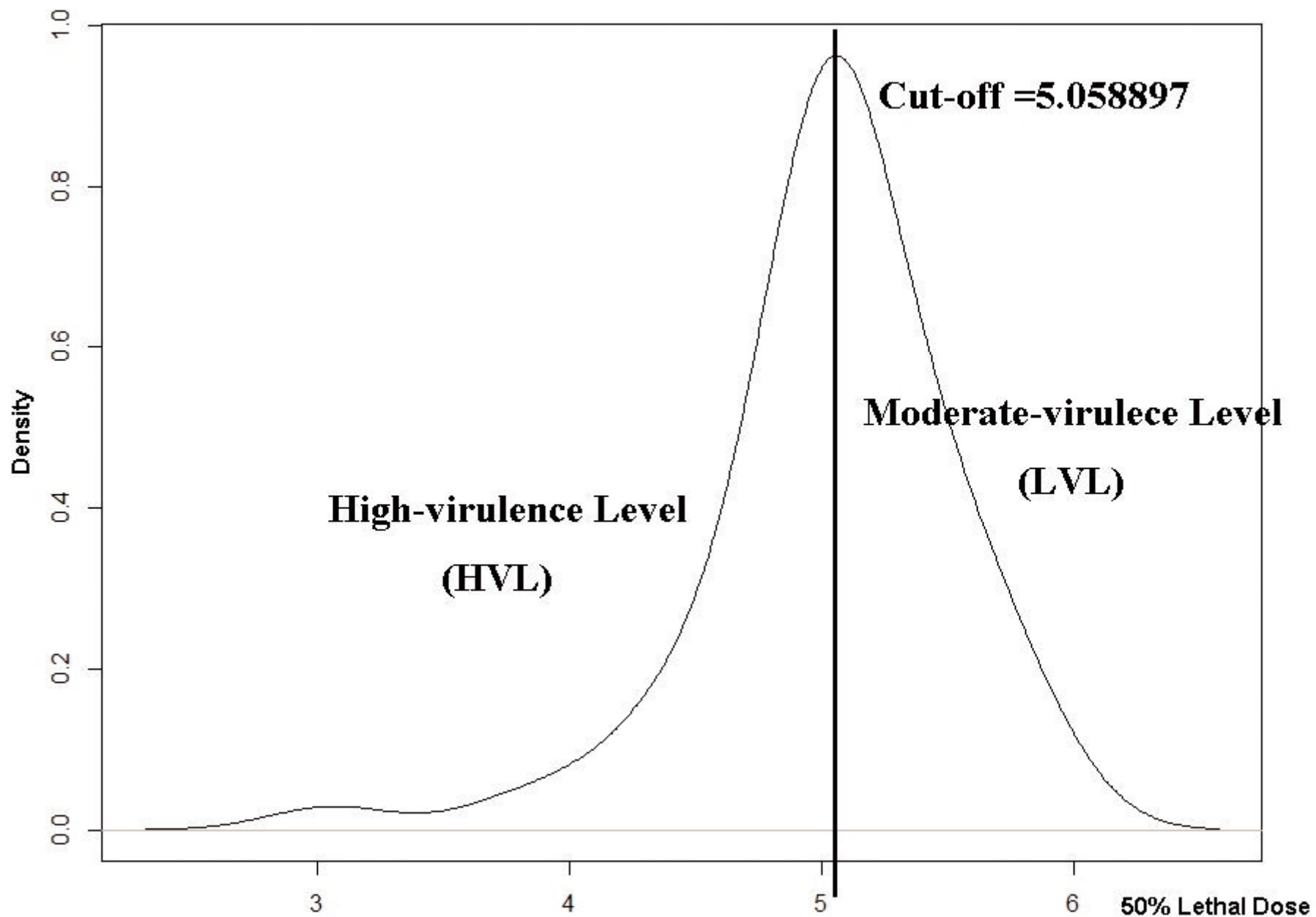

Supplement: Supplementary file 1 — Figure S1. The density graph of LD50 of 56 hypervirulent Klebsiella pneumoniae isolates. (PDF 481 kb) [file 12866_2018_1236_MOESM1_ESM.pdf]
