# Supplementary material for: Diversity of virulence level phenotype of hypervirulent Klebsiella pneumoniae from different sequence type lineage
Source: BMC Microbiol. 2018 Aug 29;18:94. doi: 10.1186/s12866-018-1236-2 (PMC6116568; doi:10.1186/s12866-018-1236-2)
Supplement: Supplementary file 2 — Table S1. PCR primers used for studying Serotype and Virulence-Associated Genes of Klebsiella pneumoniae isolates. (PDF 481 kb) (DOCX 27 kb) [file 12866_2018_1236_MOESM2_ESM.docx]

**Table S1** **Table S1 PCR primers used for studying Serotype and Virulence-Associated Genes of** ***Klebsiella pneumoniae* isolates**

| Target gene | Primer | Reference |
| --- | --- | --- |
| K1  Forward  Reverse | *5’-GTAGGTATTGCAAGCCATGC-3’*  *5’-GCCCAGGTTAATGAATCCGT-3’* | (Li et al., 2014) |
| K2  Forward  Reverse | *5’-GGAGCCATTTGAATTCGGTG-3’*  *5’-TCCCTAGCACTGGCTTAAGT-3’* | (Li et al., 2014) |
| *magA*  Forward  Reverse | *5’-GGTGCTCTTTACATCATTGC-3’*  *5’-GCAATGGCCATTTGCGTTAG-3’* | (Fang et al., 2004) |
| *rmpA*  Forward  Reverse | *5’-ACTGGGCTACCTCTGCTTCA-3’*  *5’-CTTGCATGAGCCATCTTTCA-3’* | (Yu et al., 2006) |
| *kfu*  kfuB-F  kfuC-R | *5’-GAAGTGACGCTGTTTCTGGC-3’*  *5’-TTTCGTGTGGCCAGTGACTC-3’* | (Ma et al., 2005) |
| *fimH*  Forward  Reverse | *5’-TGCTGCTGGGCTGGTCGATG-3’*  *5’-GGGAGGGTGACGGTGACATC-3’* | (Yu et al., 2006) |
| *wabG*  Forward  Reverse | *5’-ACCATCGGCCATTTGATAGA-3’*  *5’-CGGACTGGCAGATCCATATC-3’* | (Izquierdo et al., 2003) |
| *uge*  Forward  Reverse | *5’-TCTTCACGCCTTCCTTCACT-3’*  *5’-GATCATCCGGTCTCCCTGTA-3‘* | (Regue et al., 2004) |
| *iroN*  Forward  Reverse | *5’-AAGTCAAAGCAGGGGTTGCCCG-3’*  *5’-GACGCCGACATTAAGACGCAG-3’* | (Mamlouk et al., 2006) |
| *iutA*  Forward  Reverse | *5’-GGCTGGACATCATGGGAACTGG-3’*  *5’-CGTCGGGAACGGGTAGAATCG-3’* | (Mamlouk et al., 2006) |
| *allS*  Forward  Reverse | *5’-CCGAAACATTACGCACCTTT-3’*  *5’-ATCACGAAGAGCCAGGTCAC-3’* | (Luo et al., 2014) |
| *entB*  Forward  Reverse | *5’-ATTTCCTCAACTTCTGGGGC-3’*  *5’-AGCATCGGTGGCGGTGGTCA-3’* | (Candan and Aksöz, 2015) |

**References**

Candan, E. D., and Aksöz, N. (2015). *Klebsiella pneumoniae* : characteristics of carbapenem resistance and virulence factors. *Acta Biochim. Pol.* 62, 3–10. doi:10.18388/abp.2015_1148.

Fang, C.-T., Chuang, Y.-P., Shun, C.-T., Chang, S.-C., and Wang, J.-T. (2004). A Novel Virulence Gene in *Klebsiella pneumoniae* Strains Causing Primary Liver Abscess and Septic Metastatic Complications. *J. Exp. Med.* 199, 697–705. doi:10.1084/jem.20030857.

Izquierdo, L., Coderch, N., Pique, N., Bedini, E., Corsaro, M. M., Merino, S., et al. (2003). The *Klebsiella pneumoniae wabG* Gene: Role in Biosynthesis of the Core Lipopolysaccharide and Virulence. *J. Bacteriol.* 185, 7213–7221. doi:10.1128/JB.185.24.7213-7221.2003.

Li, W., Sun, G., Yu, Y., Li, N., Chen, M., Jin, R., et al. (2014). Increasing occurrence of antimicrobial-resistant hypervirulent (Hypermucoviscous) *Klebsiella pneumoniae* isolates in China. *Clin. Infect. Dis.* 58, 225–232. doi:10.1093/cid/cit675.

Luo, Y., Wang, Y., Ye, L., and Yang, J. (2014). Molecular epidemiology and virulence factors of pyogenic liver abscess causing *Klebsiella pneumoniae* in China. *Clin. Microbiol. Infect.* 20, O818–O824. doi:10.1111/1469-0691.12664.

Ma, L.-C., Fang, C.-T., Lee, C.-Z., Shun, C.-T., and Wang, J.-T. (2005). Genomic heterogeneity in *Klebsiella pneumoniae* strains is associated with primary pyogenic liver abscess and metastatic infection. *J. Infect. Dis.* 192, 117–128. doi:10.1086/430619.

Mamlouk, K., Boubaker, I. B. B., Gautier, V., Vimont, S., Picard, B., Ben Redjeb, S., et al. (2006). Emergence and outbreaks of CTX-M β-lactamase-producing *Escherichia coli* and *Klebsiella pneumoniae* strains in a Tunisian hospital. *J. Clin. Microbiol.* 44, 4049–4056. doi:10.1128/JCM.01076-06.

Regue, M., Hita, B., Pique, N., Izquierdo, L., Merino, S., Fresno, S., et al. (2004). A Gene, uge, Is Essential for *Klebsiella pneumoniae* Virulence. *Infect. Immun.* 72, 54–61. doi:10.1128/IAI.72.1.54-61.2004.

Yu, W.-L., Ko, W.-C., Cheng, K.-C., Lee, H.-C., Ke, D.-S., Lee, C.-C., et al. (2006). Association between rmpA and magA Genes and Clinical Syndromes Caused by *Klebsiella pneumoniae* in Taiwan. *Clin. Infect. Dis.* 42, 1351–1358. doi:10.1086/503420.
